# Association between the levels of urine kidney injury molecule-1 and the progression of acute kidney injury in the elderly

**DOI:** 10.1371/journal.pone.0171076

**Published:** 2017-02-10

**Authors:** Yuanyuan Xie, Qin Wang, Chunlin Wang, Xiajing Che, Xinghua Shao, Yao Xu, Zhaohui Ni, Shan Mou

**Affiliations:** 1 Department of Nephrology, Molecular Cell Laboratory for Kidney Disease, Ren Ji Hospital, School of Medicine, Shanghai Jiao Tong University, Shanghai, P.R. China; 2 Shanghai Center for Peritoneal Dialysis Research, Shanghai, P.R. China; University of Leicester, UNITED KINGDOM

## Abstract

**Background:**

The factors influencing the prognosis of acute kidney injury (AKI) were analyzed in a group of elderly AKI patients to determine the markers of early prognosis.

**Methods:**

A total of 258 patients were screened, and 201 patients were enrolled in the study. Eventually, 184 AKI patients were included in the study, including 79 elderly AKI patients (≥60 years old). During one year of follow-up, renal function changes were observed, and the risk factors that influenced the prognosis of AKI were analyzed.

**Results:**

When AKI occurred, the urine kidney injury molecule-1 (uKIM-1) level was significantly higher in the progressive deterioration of renal function group than in the renal function stable group. The ROC curve analysis revealed that the area under the curve for poor progressive deterioration of renal function as predicted by the uKIM-1 level was 0.681. At a cutoff point of 2.46 ng/mg, the sensitivity was 71.9% and the specificity was 70.0%. In elderly AKI patients, uKIM-1 levels exceeding 2.46 ng/mg were positively associated with poor kidney prognosis.

**Conclusions:**

Elderly AKI patients are at risk of developing progressive deterioration of renal function. In elderly AKI patients, the high uKIM-1 level may predict the prognosis of kidney function and may be used as an early screening indicator of poor kidney prognosis.

## Introduction

Among inpatients, acute kidney injury (AKI) is a common complication, with a high incidence and poor prognosis. AKI consumes a large amount of societal medical resources [1]. Provide specific therapies for the cause of AKI. Apply renal replacement therapy if necessary. In recent years, more studies have concentrated on the relationship between AKI and chronic kidney disease (CKD). Compared with young patients, elderly AKI patients can easily progress to CKD or end-stage renal disease (ESRD) [2–4]. Urinary kidney injury molecule-1 (uKIM-1) is a marker of epithelial injury of renal tubules, and it is elevated in the early stages of AKI [5]. Different uKIM-1 levels are associated with various degrees of renal injuries. For this study, the factors influencing kidney prognosis were analyzed in a group of elderly AKI patients to determine the markers of early prognosis.

## Subjects and methods

### 1.1 Study subjects

Male or female inpatients (≥18 years old) with complete clinical data and who were diagnosed with AKI or acute-on-chronic kidney injury (A on C) between April 2013 and April 2015 were enrolled in this study. Patients with a life expectancy of less than one year due to malignant diseases were excluded.

AKI was defined and staged according to the Kidney Disease Improving Global Outcomes (KDIGO) Clinical Practice Guidelines for Acute Kidney Injury[6].

The study was approved by the Ethics Committee of Renji Hospital, School of Medicine, Shanghai Jiaotong University and all participants gave written informed consent.

### 1.2 Sampling

Fresh urine and blood samples were obtained from each patient at the time of the final AKI diagnosis, at 24 h and 48 h after the diagnosis, and at the three-month, six-month and twelve-month follow-ups. The samples were submitted to a biochemical laboratory for testing.

First, 10 mL fresh urine was obtained from each patient at the final AKI diagnosis, and the urine was centrifuged at 1000×g for 15 min. Then, supernatant was placed into an Eppendorf tube and preserved at -80°C until testing.

### 1.3 Detection of indicators

Serum creatinine (sCr) and urine creatinine (uCr) levels were analyzed using enzymatic methods. A simplified Modification of Diet in Renal Disease (MDRD) formula was adopted to estimate the glomerular filtration rate (GFR), eGFR = 186×(sCr/88.4)^-1.154^×age^-0.203^X(0.742, female) [7].

A sandwich enzyme linked immunosorbent assay (ELISA) was performed to detect urine kidney injury molecule-1 (uKIM-1) using a kit produced by the American R&D Corporation (Minneapolis, USA). The concentration of the tested sample (undiluted urine) was calculated using the standard curve and expressed in ng/mg after synchronous uCr correction.

### 1.4 Grouping

Patients ≥60 years old were divided into a transient AKI group and a non transient AKI group based on the changes in renal function. Transient AKI was defined as reversal within 48 h[8].

During the one year of follow-up, renal function changes were observed, and the risk factors for progressive deterioration of renal function were analyzed. Progressive deterioration of renal function is defined as a one-stage drop in GFR, accompanied by a 25% or greater drop in eGFR compared with baseline [9].

### 1.5 Statistical analyses

SPSS13.0 statistical software was used for the analyses. Normal distribution measurement data are expressed as x±s, and a t-test was used for between-group comparisons. Non-normally distributed data are expressed as the median (M) and interquartile range (P25, P75), and the Wilcoxon rank-sum test was used for between-group comparisons. The ROC curve and the area under curve were adopted to calculate the sensitivity and specificity, respectively. Logistic regression was used to analyze the related risk factors that influenced kidney prognosis. Cox multifactor regression analysis was used to investigate the relationship between all risk factors and the life span of the kidney. The patient survival rate was analyzed using the Kaplan-Meier life survival curve, and a P<0.05 difference was considered to be statistically significant.

## Results

### 2.1 General information

**Fig 1:**
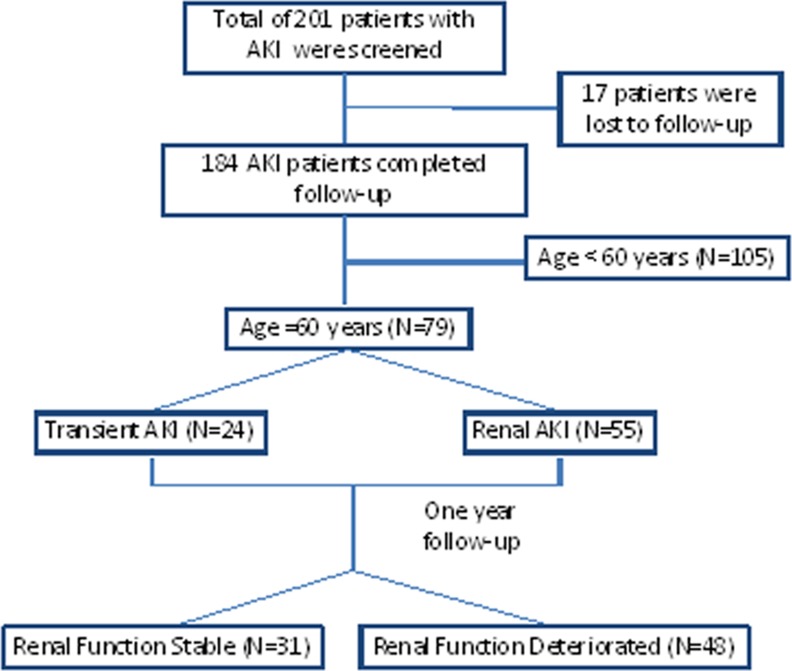
Study flow chart.

A total of 258 patients were screened, and 201 patients were enrolled in the study. Seventeen patients were excluded due to loss of follow-up. Eventually, 184 AKI patients were enrolled in the study, including 79 patients ≥60 years old and 105 patients <60 years old (Fig 1).

These 184 AKI patients (age range: 18 to 99 years) had a median age of 53.00 years (31.00, 64.75), with a 1.75:1 ratio of males to females. The mean follow-up period was 12.2±1.06 months. The baseline Scr, baseline eGFP and baseline 24 h total urinary protein levels were 98.87 μmol/L (76.85, 211.85), 66.68 mL/min/1.73 m^2^ (28.10, 94.18)and 3.78 g (1.47, 9.08), respectively. A total of 71 patients had a previous history of chronic renal disease. In AKI, the uKIM-1 level and peak creatinine values were 2.37 ng/mg (1.10, 6.22) and 215.00 μmol/L (124.40, 331.00), respectively. Stage 1 disease was found in 123 patients, stage 2 was found in 26 patients, and stage 3 was found in 35 patients. The incidence of non transient AKI was 53.26%. The etiologies of AKI were assessed clinically, including renal blood hypoperfusion or ischemia (22%), renal toxicity injury (20%), infectious factors (23%), renal glomerular disease aggravation or activity (19%), and obstruction (11%). After three months of follow-up, renal functional rehabilitation was observed in 59.78% of the patients. After one year of follow-up, progressive deterioration of renal function was found in 39.67% of the patients (Table 1).

**Table 1:** General condition of patients.

Characteristics	N = 184
Age, years	53.00(31.00, 64.75)
Male sex (N, %)	117(63.59%)
Oliguria (N, %)	9(4.9%)
Baseline serum creatinine (μmol/l)	98.87(76.85, 211.85)
Baseline estimated GFR(ml/min/1.73m^2^)	66.68(28.10, 94.18)
Baseline 24hUTP(g/24h)	3.78(1.47, 9.08)
sAlb(g/l)	33.30(24.00, 40.25)
Hb(g/L)	119.00(99.25, 140.00)
ALT(IU/L)	14.00(10.00, 23.00)
AST(U/L)	17.00(14.00, 25.75)
Ca(mmol/L)	2.08(1.99, 2.22)
P(mmol/L)	1.32(1.10, 1.56)
K(mmol/L)	3.90(3.40, 4.30)
HCO_3_^-^(mmol/L)	24.20(20.90, 26.40)
hsCRP(mg/L)	2.40(0.80, 8.17)
*Cause of AKI*	*N(%)*
ischemia	40(21.74%)
Nephrotoxin exposure	37(20.11%)
Infection	42(22.83%)
Glomerular disease	35(19.02%)
obstruction	21(11.41%)
others	9(4.90%)
*AKI stage*	
Stage 1	123
Stage 2	26
Stage 3	35
Peak serum creatinine (μmol/l)	215.00(124.40, 331.00)
uKIM-1(ng/mg)	2.37(1.10, 6.22)
FeNa≥1%	126(68.48%)
RFI≥1%	136(73.91%)
non transient AKI	98(53.26%)
Recovery within 3 months	110(59.78%)
Ultimate outcome	73(39.67%)

### 2.2 Condition analysis for elderly AKI patients

Comparing AKI patients ≥60 years old (N = 79) with patients <60 years old (N = 105), no significant differences were observed in sex, baseline creatinine level, proportion of pre-existing chronic renal disease and AKI staging. The etiologies of AKI in elderly patients primarily involved renal blood hypoperfusion or ischemia, renal toxicity injury and infections.

Elderly AKI patients ≥60 years old presented with low baseline eGFR levels and higher peak values of creatinine during the occurrence of AKI. Patients with FeNa≥1% and RFI≥1% accounted for a higher proportion of patients, and the incidence of non transient AKI was higher in these patients.

For the elderly AKI patients ≥60 years old, higher uKIM-1 levels were observed during the occurrence of AKI, but no significant difference was identified between the elderly AKI patients (≥60 years old) and those AKI patients who aged from 18 to 60.

For patients ≥60 years of age, renal functional rehabilitation was observed in 45.57% patients within three months after being diagnosed with AKI, which was significant compared with patients <60 years old (70.48%). After the one-year follow-up period, progressive deterioration of renal function was observed in 60.76% of the patients ≥60 years old, a rate that was significantly higher than that of patients <60 years old (23.81%) (Table 2).

**Table 2:** Comparison between different age groups.

Characteristics	≥60 years (N = 79)	<60 years (N = 105)	P-value
Age, years	69.00(64.25, 77.00)	39.50(28.00, 53.00)	0.000
Male sex (N, %)	48(60.76%)	69(65.71%)	0.537
Oliguria (N, %)	5(6.33%)	4(3.81%)	0.501
Baseline serum creatinine (μmol/L)	129.60(82.53, 252.90)	95.50(74.30, 161.00)	0.082
Baseline estimated GFR(mL/min/1.73 m^2^)	44.32(19.75, 83.76)	77.70(39.72, 104.63)	0.011
Baseline 24 h UTP	2.47(0.53, 9.09)	3.82(1.89, 9.11)	0.261
sAlb(g/L)	33.00(25.30, 37.40)	33.35(22.00, 40.55)	0.749
Hb(g/L)	102.50(84.25, 127.75)	125.00(105.50, 143.00)	0.000
ALT(IU/L)	11.00(8.00, 20.00)	15.00(10.00, 28.25)	0.029
AST(U/L)	17.00(14.00, 22.50)	17.50(14.00, 26.25)	0.411
Ca(mmol/L)	2.03(1.87, 2.20)	2.11(1.88, 2.22)	0.467
P(mmol/L)	1.34(1.08, 1.69)	1.32(1.10, 1.54)	0.668
K(mmol/L)	3.90(3.40, 4.40)	3.80(3.40, 4.20)	0.735
HCO_3_^-^(mmol/L)	24.10(20.40, 26.90)	24.40(21.45, 26.40)	0.471
hsCRP(mg/L)	7.38(1.98, 28.70)	1.68(0.53, 4.32)	0.000
*Cause of AKI*			*0*.*886*
Ischemia	18(22.78%)	22(20.95%)	
Nephrotoxin exposure	16(20.25%)	21(20.00%)	
Infection	19(24.05%)	23(21.90%)	
Glomerular disease	12(15.19%)	23(21.90%)	
Obstruction	9(11.39%)	12(11.43%)	
Other	5(6.33%)	4(3.81%)	
*AKI stage*			0.603
Stage 1	50	73	
Stage 2	14	12	
Stage 3	15	20	
Peak serum creatinine (μmol/L)	285.35(148.50, 386.03)	191.00(117.35, 301.70)	0.006
uKIM-1(ng/mg)	2.75(1.68, 7.44)	1.76(0.77, 6.07)	0.055
FeNa≥1%	68(86.08%)	58(55.24%)	0.000
RFI≥1%	69(87.34%)	67(63.81%)	0.000
non transient AKI	55(69.62%)	43(40.95%)	0.000
Recovery within three months	36(45.57%)	74(70.48%)	0.001
Ultimate outcome	48(60.76%)	25(23.81%)	0.000

### 2.3 Analysis of factors related to poor kidney prognosis in elderly AKI patients

Among the 79 AKI patients ≥60 years old, the transient AKI group included 24 patients and the non transient AKI group included 55 patients. After one year of regular follow-up, the renal function stable group included 31 patients, and the progressive deterioration of renal function included 48 patients (Fig 1).

#### 2.3.1 Comparison between the transient AKI and non transient AKI groups

**Fig 2:**
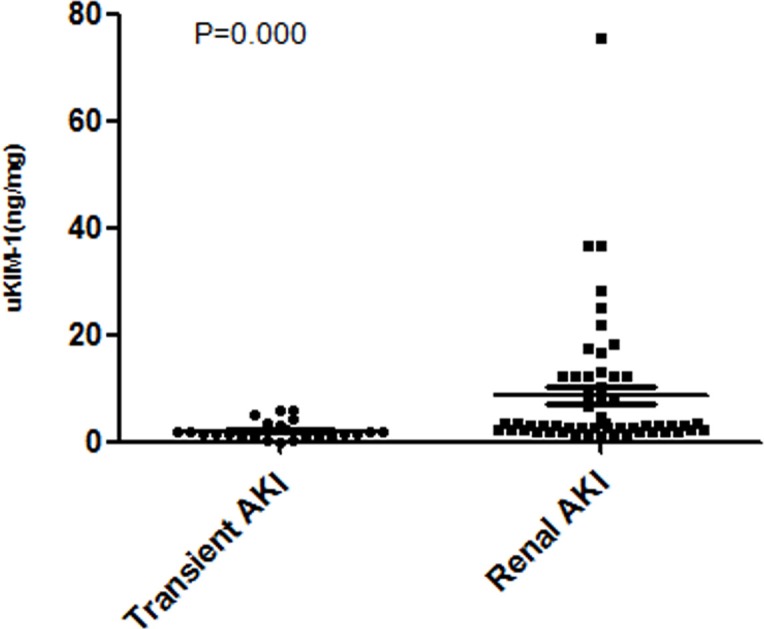
The uKIM-1 level in the transient AKI group was significantly higher than that of the non transient AKI group among elderly AKI patients during the occurrence of AKI.

No differences in age or sex were observed between two groups. After AKI was diagnosed, the peak value of creatinine in the transient AKI group was lower than that in the non transient AKI group, but no significant difference was observed. The uKIM-1 level during the occurrence of AKI in the transient AKI group was significantly lower than that in the non transient AKI group (Table 3, Fig 2).

**Table 3:** Comparison between the transient AKI group and the non transient AKI group among elderly patients with AKI.

Characteristics	Transient AKI (N = 24)	non transient AKI (N = 55)	P
Age, years	69.00(63.50, 77.00)	69.00(65.00, 78.00)	0.748
Male sex (N, %)	15(62.5%)	33(60.00%)	1.000
Peak serum creatinine (μmol/L)	183.00(118.98, 340.50)	288.90(195.60, 395.60)	0.148
uKIM-1(ng/mg)	1.56(0.72, 2.80)	3.14(2.32, 12.17)	0.000

According to logistic regression analysis, the uKIM-1 level was a risk factor for non transient AKI (Table 4).

**Table 4:** Logistic regression shows that the uKIM-1 level is a risk factor for non transient AKI.

	OR(CI 95%)	P
Age		0.406
Sex		0.645
uKIM-1(ng/mg)	1.579(1.037, 2.404)	0.033

**Fig 3:**
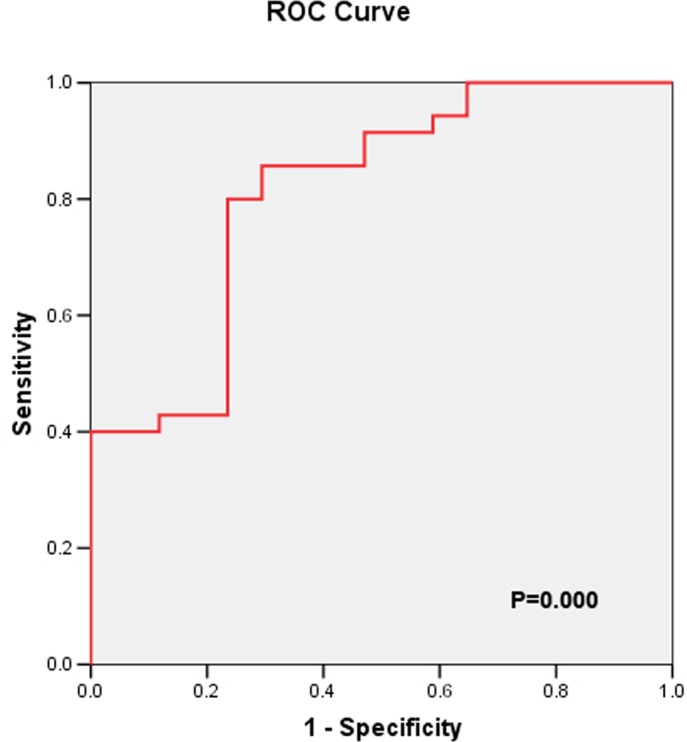
ROC-AUC of non transient AKI according to uKIM-1 level.

Further ROC curve analysis indicated that the area under the curve for non transient AKI according to the uKIM-1 level was 0.812. When the cutoff point was 2.13 ng/mg, the sensitivity and specificity were 80.0% and 76.5%, respectively (Fig 3).

#### 2.3.2 Comparison between the renal function stable and progressive deterioration of renal function groups

After one year of regular follow-up in elderly AKI patients, no significant differences were observed in the age, sex, and baseline 24 h urine protein quantity between the renal function stable group and progressive deterioration of renal function group.

**Fig 4:**
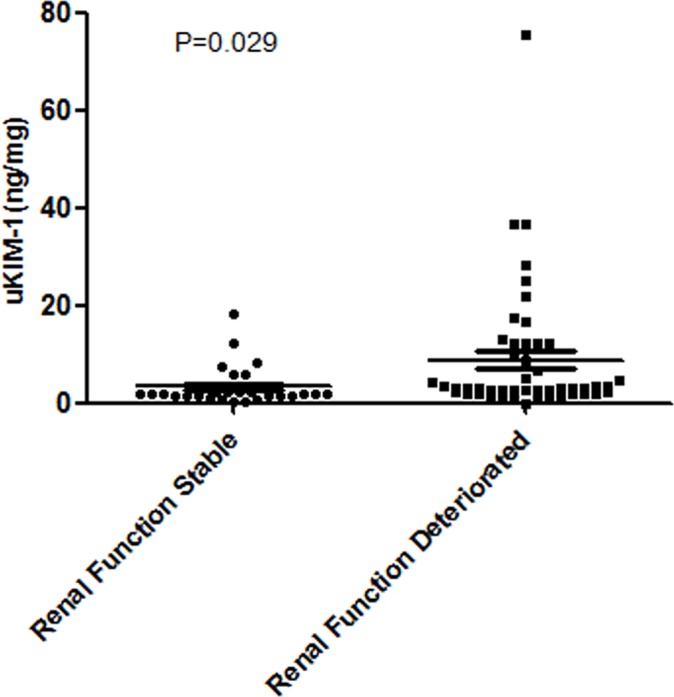
The uKIM-1 level of the progressive deterioration of renal function group was significantly higher than that of the renal function stable group among elderly patients with AKI.

The baseline creatinine level in the progressive deterioration of renal function group was significantly higher than that of the stable renal function group, whereas the baseline eGFR level in the progressive deterioration of renal function group was significantly lower than that of the stable renal function group. During AKI, significantly higher uKIM-1 levels and peak serum creatinine values were observed in the progression group compared with the stable group (Table 5, Fig 4).

**Table 5:** Comparison between the progressive deterioration of renal function group and the renal function stable group after the one year of follow-up for elderly patients with AKI.

Characteristics	Renal Function Stable (N = 31)	Progressive deterioration of renal function (N = 48)	P
Age, years	68.00(64.50, 73.50)	71.00(64.50, 77.50)	0.286
Male sex (N, %)	20(64.52%)	28(58.33%)	0.642
Baseline serum creatinine (μmolLl)	93.80(64.00, 203.70)	187.00(87.85, 277.70)	0.038
Baseline estimated GFR(mL/min/1.73 m^2^)	74.43(26.57, 91.21)	31.95(17.58, 62.57)	0.022
Baseline 24 h UTP(g/24 h)	2.75(0.79, 16.31)	2.19(0.48, 8.34)	0.703
Peak serum creatinine (μmol/L)	174.00(118.95, 302.45)	331.35(214.85, 398.30)	0.011
uKIM-1(ng/mg)	2.02(1.43, 3.30)	3.25(1.89, 11.70)	0.029

Multifactor Cox regression analysis showed that the uKIM-1 level was an independent risk factor for progressive deterioration of renal function (Table 6).

**Table 6:** Cox regression shows that uKIM-1 is an independent risk factor for progressive deterioration of renal function.

	RR (CI 95%)	P
Age		0.339
Sex		0.130
Peak serum creatinine (μmol/L)		0.173
uKIM-1(ng/mg)	1.026(1.003, 1.050)	0.026

**Fig 5:**
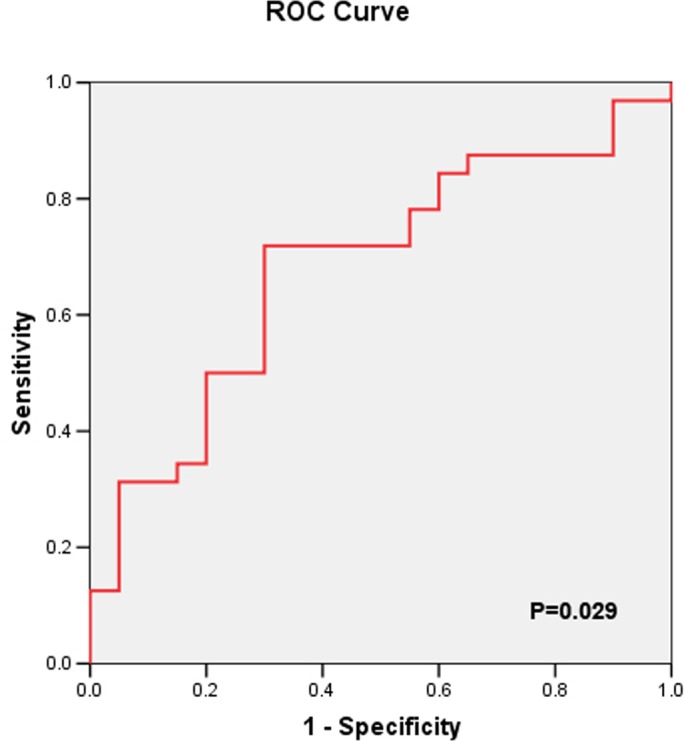
ROC-AUC of progressive deterioration of renal function predicted by uKIM-1 level.

Additional ROC curve analysis indicated that the area under the curve for progressive deterioration of renal function as determined by the uKIM-1 level was 0.681. When the cutoff point was 2.46 ng/mg, the sensitivity and specificity were 71.9% and 70.0%, respectively (Fig 5).

**Fig 6:**
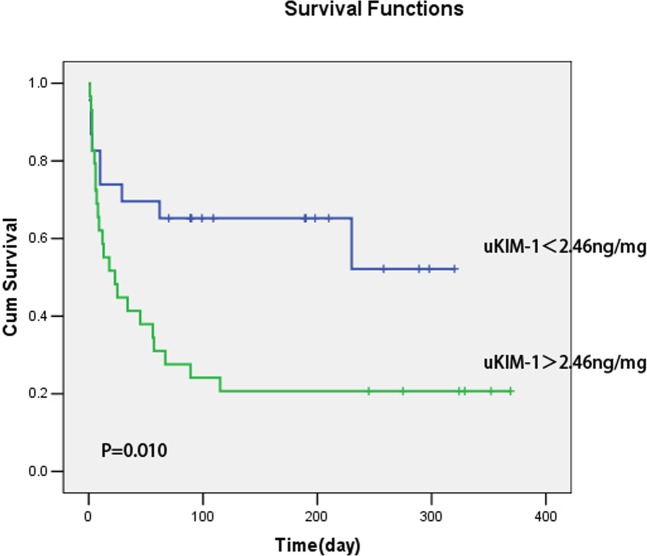
The uKIM-1 level and kidney prognosis. A uKIM-1 level > 2.46 ng/mg is positively related to poor prognosis.

A uKIM-1 level > 2.46 ng/mg in elderly AKI patients was positively correlated with poor kidney prognosis (Fig 6).

## Discussion

AKI is a common clinical syndrome that primarily presents with a rapid decrease in renal function and an accumulation of metabolic waste. For many years, AKI was considered to be a self-limiting disease. However, in recent years, the incidence of CKD has gradually increased in conjunction with an increased incidence of AKI. More studies have reported that some AKI patients do not completely recover and gradually progress to CKD and ESRD, with some patients even requiring permanent kidney replacement therapy [10, 11]. Research data indicated that CKD progression may be influenced by AKI [12, 13]. A total of 184 AKI inpatients were enrolled into the current study, and after one year of regular follow-up, progressive deterioration of renal function was observed in 40% patients. The results demonstrated that AKI may lead to CKD, which is consistent with the results of previous studies [14]. It is still unknown why AKI increases the risks of CKD and ESRD. Animal experiments have demonstrated [15] that AKI can lead to kidney fibrosis while concurrently damaging the lungs, heart, and liver. Although AKI is generally reversible, in the event of serum creatinine concentration recovery there may be subclinical damage to the kidney and extra-renal organs. This damage will continue, perhaps leading to CKD and even ESRD.

This study observed poor baseline renal function in elderly AKI patients (≥60 years old), and a higher proportion of renal AKI was observed in patients with poorer prognoses. One study that investigated the prognosis of acute interstitial nephritis in elderly patients reported [16] that histologically, elderly patients have a higher proportion of glomerulosclerosis, renal interstitial fibrosis and renal tubule atrophy. Elderly patients easily developed AKI cases that more easily progressed to CKD.

Previous studies suggested [17] that the uKIM-1 level is a good biomarker for AKI diagnosis. Nejat et al indicated that [18] KIM-1 is elevated in pre-renal AKI (AKI caused by compromised renal perfusion) and significantly elevated in renal AKI. However, no systematic verification of biomarkers was performed for elderly AKI patients [2]. This study performed a further analysis of elderly AKI patients, and the results indicated that the uKIM-1 level of the non transient AKI group was significantly higher than that of the transient AKI group during the occurrence of AKI. The uKIM-1 level is a risk factor for non transient AKI, and the sensitivity and specificity of uKIM-1 to identify non transient AKI are 80.0% and 76.5%, respectively.

van Timmeren et al examined renal biopsy samples from various renal diseases (n = 102) patients that proximal tubular cells from all patients secreted KIM-1 and that tissue KIM-1 expression was correlated with inflammation. uKIM-1 could reflect the KIM-1 level in tissues and correlate with renal interstitial inflammation and renal function. So Van Timmeren et al [19] suggested that KIM-1 is not only a biomarker of acute renal proximal tubule injury but also a marker of renal tubulointerstitial chronic inflammation and fibrosis. Previous studies confirmed [20] that uKIM-1 can effectively predict kidney prognosis. In this study, one year of regular follow-up was performed for elderly AKI patients, and the results revealed that the uKIM-1 levels of patients with progressive deterioration of renal function were significantly higher than the levels in the renal function stable group of patients. Multifactor Cox regression analysis demonstrated that the uKIM-1 level is an independent risk factor for progressive deterioration of renal function, and when the uKIM-1 level increased by 1, the risk of progressive deterioration of renal function after one year increased by 2.6%. The sensitivity and specificity of the uKIM-1 level to predict progressive deterioration of renal function were 71.9% and 70.0%, respectively. A uKIM-1 level >2.46 ng/mg in elderly AKI patients was positively related with poor kidney prognosis.

This single-center study used a small sample size. To further validate the study results, multicenter studies with larger sample sizes are required. In addition, the analysis in this study could not correct for the severity of pre-existing CKD. More advanced CKD is a strong risk factor for AKI. Hsu et al reported that [21] progressive deterioration of renal function may be related to advanced CKD but less associated with AKI.

## Conclusions

Elderly AKI patients are at risk of progressive deterioration of renal function. uKIM-1 is a good biomarker of AKI in elderly patients, and uKIM-1 levels can effectively distinguish between transient AKI and non transient AKI. In addition, uKIM-1 levels accurately predict the kidney prognosis in elderly AKI patients and may be used as an early screening indicator of poor kidney prognosis. The results of this study can help to develop new treatment strategies to assist clinical physicians in providing earlier interventions to mitigate renal progression after AKI. The renal functions of elderly patients with a history of AKI should be closely monitored.

## References

[1] Siew ED, Davenport A. The growth of acute kidney injury: a rising tide or just closer attention to detail? Kidney Int. 2015; 87:46–61. 10.1038/ki.2014.293 25229340 PMC4281297

[2] Anderson S, Eldadah B, Halter JB, Hazzard WR, Himmelfarb J, Horne FM, et al Acute kidney injury in older adults. J Am Soc Nephrol. 2011; 22:28–38. 10.1681/ASN.2010090934 21209252

[3] Ishani A, Xue JL, Himmelfarb J, Eggers PW, Kimmel PL, Molitoris BA, et al Acute kidney injury increases risk of ESRD among elderly. J Am Soc Nephrol. 2009; 20: 223–228. 10.1681/ASN.2007080837 19020007 PMC2615732

[4] Coca SG. Acute kidney injury in elderly persons. Am J Kidney Dis. 2010; 56: 122–131. 10.1053/j.ajkd.2009.12.034 20346560 PMC2902696

[5] Siew ED, Ware LB, Ikizler TA. Biological markers of acute kidney injury. J Am Soc Nephrol, 2011; 22: 810–20. 10.1681/ASN.2010080796 21493774

[6] Kidney Disease Improving Global Outcomes AKI Guideline Work Group. KDIGO Clinical Practice Guideline for Acute Kidney Injury. Kidney Int Suppl. 2012; 2: 1–138.

[7] Levey AS, Coresh J, Greene T, et al Expressing the Modification of Diet in Renal Disease Study Equation for Estimating Glomerular Filtration Rate with Standardized Serum Creatinine Values. Clin. Chem, 2007, 53:766–772. 10.1373/clinchem.2006.077180 17332152

[8] Doi K, Katagiri D, Negishi K, Hasegawa S, Hamasaki Y, Fujita T, et al Mild elevation of urinary biomarkers in prerenal acute kidney injury. Kidney Int. 2012; 82:1114–1120. 10.1038/ki.2012.266 22854644

[9] The Kidney Disease: Improving Global Outcomes (KDIGO) organization. KDIGO Clinical Practice Guideline for the Evaluation and Management of Chronic Kidney Disease. Kidney inter., Suppl. 2013; 3: 1–150.

[10] Siew ED, Davenport A. The growth of acute kidney injury: a rising tide or just closer attention to detail? Kidney Int. 2015; 87:46–61. 10.1038/ki.2014.293 25229340 PMC4281297

[11] Leung KC, Tonelli M, James MT. Chronic kidney disease following acute kidney injury-risk and outcomes. Nat Rev Nephrol. 2013;9:77–85. 10.1038/nrneph.2012.280 23247572

[12] Chawla LS, Kimmel PL. Acute kidney injury and chronic kidney disease: an integrated clinical syndrome. Kidney Int. 2012;82:516–524. 10.1038/ki.2012.208 22673882

[13] Li L, Astor BC, Lewis J, Hu B, Appel LJ, Lipkowitz MS, et al Longitudinal progression trajectory of GFR among patients with CKD. Am J Kidney Dis. 2012;59:504–512. 10.1053/j.ajkd.2011.12.009 22284441 PMC3312980

[14] Coca SG, Singanamala S, Parikh CR. Chronic kidney disease after acute kidney injury: a systematic review and meta-analysis. Kidney Int. 2012;81:442–448. 10.1038/ki.2011.379 22113526 PMC3788581

[15] Kelly KJ. Distant effects of experimental renal ischemia/reperfusion injury. J Am Soc Nephrol 2003; 14: 1549–1558. 12761255 10.1097/01.asn.0000064946.94590.46

[16] Muriithi AK, Leung N, Valeri AM, Cornell LD, Sethi S, Fidler ME, et al Clinical characteristics, causes and outcomes of acute interstitial nephritis in the elderly. Kidney Int. 2015;87:458–464. 10.1038/ki.2014.294 25185078

[17] Xue W, Xie Y, Wang Q, Xu W, Mou S, Ni Z. Diagnostic performance of urinary kidney injury molecule-1 and neutrophil gelatinase-associated lipocalin for acute kidney injury in an obstructive nephropathy patient. Nephrology.2014; 19:186–194. 10.1111/nep.12173 24165570

[18] Nejat M, Pickering JW, Devarajan P, Bonventre JV, Edelstein CL, Walker RJ, et al Some biomarkers of acute kidney injury are increased in pre-renal acute injury. Kidney Int. 2012;81:1254–1262. 10.1038/ki.2012.23 22418979 PMC3365288

[19] van Timmeren MM, van den Heuvel MC, Bailly V, Bakker SJ, van Goor H, Stegeman CA. Tubular kidney injury molecule-1 (KIM-1) in human renal disease. J Pathol, 2007, 212:209–217. 10.1002/path.2175 17471468

[20] Xie Y, Xue W, Mou S, Shao X, Che X, Ni Z, et al Analysis of a urinary biomarker panel for obstructive nephropathy and clinical outcomes. PLoS One. 2014;9:e112865 10.1371/journal.pone.0112865 25402279 PMC4234476

[21] Hsu CY, Ordonez JD, Chertow GM, Fan D, McCulloch CE, Go AS. The risk of acute renal failure in patients with chronic kidney disease. Kidney Int. 2008; 74: 101–107. 10.1038/ki.2008.107 18385668 PMC2673528

